# Exploring Correlations between Headaches and Refractive Errors in an Optometry Clinic Sample

**DOI:** 10.22599/bioj.313

**Published:** 2024-01-03

**Authors:** Samuel Otabor Wajuihian

**Affiliations:** 1 University of Kwazulu-Natal School of Health Sciences, South Africa

**Keywords:** Headaches and refractive errors, optometry, clinical setting, children and young adults

## Abstract

**Background & Aim:**

The optometrist is often one of the professionals patients consult when they have headaches. The limitations inherent in previous studies on the topic limit the utilization of their findings. Therefore, the aim of conducting the present study was to explore correlations between headache and refractive errors in a clinical setting using extended classification criteria.

**Methods:**

The study design was cross-sectional, and sample comprised (headache group = 1062; non-headache group = 1095) participants aged 10–40 years who attended an optometry practice. During case-history taking, participants were classified as headache and non-headache group. Refraction, ocular health examinations, accommodative and vergence tests were performed. Headaches were sub-classified according to the anatomic location such as temporal, frontal, occipital, or diffuse, based on where pain was felt.

**Results:**

Temporal and temporo-frontal headaches were most frequent. Participants in the *headache group* numbered 1062 with mean age 25.1 ± 8.6; females 841 (79.1%) and males 221 (20.8%) while those in the *no headache group* numbered 1095 with mean age 25.3 ± 8.7; females 648 (59.1%). Low amount spheres and cylinders (*p* = 0.003) as well as hyperopic, and against-the-rule astigmatism (*p* = 0.012) and (*p* = 0.03) respectively were significantly more frequent in the headache group.

**Conclusion:**

Temporal headaches were most frequent. Patients with low spheres and cylindrical errors as well as hyperopic and against-the-rule astigmatism were significantly more prone to headaches. This study provides findings, which have not been reported. Findings have implications for clinical practice and highlights the need to compensate for low ametropia. A standard study protocol is recommended.

## Introduction

Headaches comprise pain or discomfort, which radiate from pain-sensitive structures in the head (Bigley 1990). Based on the pain mechanism, headaches may broadly be classified as tension (muscle contraction), migraine (vascular), and clusters–types (Bigley 1990; Hale & Paaw 2014; International Classification of Headache Disorders (ICHD) 2013). Headaches may also be classified as either primary or secondary based on their aetiology (Hale & Paaw 2014; ICHD 2013). Unlike secondary headaches, primary headaches are not associated with systemic or ocular diseases (Bigley 1990; Hale & Paaw 2014; ICHD 2013). Globally, the prevalence of any headache among adult populations was estimated at 46-79% (Philipp et al. 2019). The burdens of headache are numerous. Headaches reduce health-related quality of life of their sufferers, contribute to loss of productivity and economic loss in adults, and absenteeism among school children is often linked to headaches (ICHD 2013; Philipp et al. 2019). Hence, headache has been classified as a major public-health problem worldwide (ICHD 2013; Philipp et al. 2019).

The classification Committee of the International Headache Society (CIH) described headache associated with refractive errors (HARE) as an ocular headache, usually primary, tension-type and having a functional aetiology-being attributed to performance of near point activities (ICHD 2013). Uncorrected refractive errors (URE) are the second leading cause of treatable blindness worldwide (ICHD 2013; Wajuihian & Mashige 2021). Uncorrected refractive errors impair vision, cause binocular vision anomalies and result in decreased academic and work performance (Wajuihian & Mashige 2021). The frequency of refractive errors in patients aged six to 85 years seen at an optometry clinic was 67.8% (Wajuihian & Mashige 2021). Refractive errors relate to headaches in both aetiology and treatment as baseline compensation for URE have been found to alleviate headaches (Grosvenor 2007; Wajuihian 2015). Given the anatomical link between the eye and head, patients often consult eye care professionals when they have headaches. Headache is the most frequent symptom associated with eyestrain (Donahue 1958; Mvitu & Kaimbo 2003; Wajuihian 2015; Grosvenor 2007; Cameron 1976) and among patients who consulted the eye care practitioner (Donahue 1958; Cameron 1976). The frequency of headaches in eye care practice settings range between 11.6 and 84% (Wajuihian 2022). The frequency of headaches among patients from the population from which the present study was derived is 41.1% (Wajuihian 2022).

Due to the consequences of headaches and URE, (Appendix A) the topic is of great research interest and various aspects of the topic have been explored Appendix A. For studies, which reported only frequencies, the frequency ranges of URE among headache patients are: myopia 12–35.7%, hyperopia 12-31%, and astigmatism 33–43.9%. The studies which enrolled headache and (no-headache) include Akinci et al. (2008) who retrospectively analyzed records of 310 patients aged eight to 18 years with headache and 843 patients aged seven to 17 years for control groups. Akinci et al. (2008) did not study axis astigmatism and reported that the frequency of URE was higher in the HG. In a study of 228-headache and 72-control group, Das & Gupta 2017 did not classify astigmatism according to co-existing spherical components (sphero-astigmatism). Das & Gupta 2017 found that the frequency of URE was significantly higher in the HG while Abolbashari et al. (2014) found that the URE were not significantly associated with headaches. Overall, there were differences in findings across studies, which is related to the differences in study designs.

In addition to highlighted limitations of previous studies, astigmatism types such as axis astigmatism, sphero-astigmatism, and amounts of URE which influences symptoms, visual acuity, and visual perception (Grosvenor 2007; O’Leary & Evans 2003) were rarely studied. Furthermore, cycloplegia was not applied in some studies (Appendix A), some were retrospective (Appendix A), others mostly used ambiguous criteria and classification system (Jain et al. 2015; Kondam & Settypalli 2017; Supriya & Nakkella 2021; Gil-Gouveia & Martins 2002) while sample size was relatively small in some studies (Appendix A). Interestingly, most of the previous studies were on Asian populations, published in non-optometry journals (Appendix A plus reference list) with only one available study on African populations (Al-Rasheed 2020).

Beside addressing limitations of previous studies, the present study is unique in several ways. First, no available study related refractive errors to headaches based on anatomical locations where headaches were mostly felt. Furthermore, in this study, headache and URE were sub-classified according to their severities while astigmatism was categorized according to its severity, cylinder axis orientation, as well as with spherical components. In 2007, Grosvenor highlighted the lack of clinical studies on the relation of URE and anatomic location of headaches, which Grosvenor (2007) and emphasised the need for studies on the topic.

The aim of conducting the present study, therefore, is to investigate the association between headaches and refractive errors. The specific objectives of this study were: to determine frequency of refractive errors and headaches types in relation to headache locations and explore correlations among them, and to investigate the distribution of headache types in refractive errors and correlations among them.

## Methods

### Study design and setting

This was a cross sectional study of patients seen in the author’s optometry practice in Empangeni, South Africa.

### Sample and participants

The Sample comprised: 1062 participants in the headache group, their mean age was 25.1 ± 8.6 females 841 (79.1%) and males 221 (20.8%) while those in the no-headache group were 1095, their mean age was 25.3 ± 8.7., females 648 (59.1%), The participants were Black South Africans, who attended the author’s optometry practice for routine eye care. Their records revealed that they came from 25 residential areas including rural, suburban and urban areas around the municipality.

### Eligibility criteria

Included were:

- Males & females.
- Patients aged between 10 and 40 years.

Excluded were:

- Patients who had any ocular pathology.
- Patients who were diabetic or strabismic.
- Patients who had migraine headaches.

The participants’ age range is of research interest as it embraces persons who mostly engage in more prolonged near tasks such as the use of computers and smartphones. Therefore, may be prone to functional anomalies.

### Ethics clearance

Patients signed consents on their individual practice data collection record cards after the purpose and scope of the study was described to them. The Biomedical Research Ethics Committee ((BE096) of the University of KwaZulu-Natal, South Africa approved the study protocol. Overall, the conduct of the study complied with guidelines in accordance with the Declaration of Helsinki.

### Data collection

Data were collected between January and December of 2021.The case history taking embraced records of patient-reported ocular and systemic symptoms. It was deemed unnecessary to use a precompiled symptoms questionnaire as I intended to evaluate patients based on their routine vision anomalies and associated symptoms as reported by them. All cases were treated as new even if they had glasses as presenting headaches even with their glasses might imply that the glasses were no longer effective. Furthermore, patients who consulted for follow-up examination with respect to any optical correction or vision therapy were excluded. No patient’s record was duplicated as data for all patients including those on follow-up visit were recorded only once.

Visual acuity was measured using the Snellen’s chart at 6 meters. Ocular health status was evaluated using the direct ophthalmoscope (Welch Allyn) and the Slit lamp Biomicroscope (Zeiss SL120/130). Objective refraction was done using the Welch Allyn Streak retinoscope and refined subjectively to the best visual acuity. Cycloplegic refraction was performed on all children younger than 13 years. Patients who were not cyclopleged for whatever reason were fogged with a + 2.00 D lens to screen for latent hyperopia. Astigmatic power and axis was refined using the Jackson cross cylinder.

### Headaches and other symptoms

Case history taken were records of all patients and classified as *headache (main)* and *no-headache groups*-HG and NHG respectively. Although headache is the main symptom of interest (main outcome variable), other symptoms recorded included *tearing, itchy eyes, diplopia, sandy/grittiness, photophobia, redness, tired eyes, and near blur*. Headache (ocular headache) as used in this study follows the description in the International Classification of Headache Disorders (ICHD) (ICHD 2013) which classified headache associated with refractive errors (HARE) to include: recurrent mild headache, frontal, and in the eyes themselves, which fulfils the following criteria: headache and eye pain first develop in close temporal, relation to the refractive error, are absent on awakening and aggravated by prolonged visual tasks (ICHD 2013). Such headaches may be constant or periodic, of long or short duration or may occur at regular or irregular intervals (ICHD 2013; Grosvenor 2007). Therefore, the patients enrolled included those who had consistent headaches and of a magnitude that caused much discomfort, disrupted performance of usual near point activities, and warranted patients consulting the optometrist. It is noteworthy that patients often use the terms ‘headaches’ and ‘pains’ interchangeably (Gordon 1966) where, for example, pain on the temple will be same as temporal headaches.

Patients with migraine headaches were excluded as its aetiological mechanism of action differs from other headache types (ICHD 2013). Furthermore, to extend the study variables of interest, the headache types were subclassified according to the anatomical locations or topography where the pain is felt. This sub-classification included temporal headache (TH), frontal headache (FH), occipital headache (OH), and diffuse headache (DH). There was no record of parietal headache as no patients reported on parietal headache location and this headache type is not commonly reported in the literature therefore was not included in the present study. Records of the intensity and duration of headaches were not analyzed as the patients’ responses were inconsistent.

### Diagnostic criteria and classifications of outcome variables

Refractive status was classified based on (Table 1) (Wajuihian & Mashige 2021). Absolute values (not spherical equivalent) of refraction were used for analysis.

**Table 1:** Diagnostic criteria for myopia, hyperopia, anisometropia and astigmatism.


CRITERIA APPLIED TO DEFINE REFRACTIVE ERRORS AND CATEGORIES

**Myopia (≤–0.50 DS)**

Mild: 0.5 to 3.0 DS

Moderate: 3.25 to 6.0 DS

High: >6.25 DS

**Hyperopia (≥0.50 DS)**

Mild: 0.50 to 2.0 DS

Moderate: 2.25 to 4.00 DS

High: ≥4.25 DS

**Anisometropia [(± 0.75 or 0.75 DC minus cyl)** difference between both eyes**)]**

low: 0.75 to 2

Moderate: 2.25 to 3

High: >3

**Astigmatism ≥ / –0.75/DC**

Astigmatism is presented in negative power notation and was further categorized as:

a) *Magnitude-astigmatism*

Low astigmatism (LA): 0.25 to 0.50 DC

Moderate astigmatism (MA): 0.75 to 2 DC

High astigmatism (HA): >2.00 DC

b) *Axis astigmatism*

Any amount of astigmatism- with With-the-rule (WTR), Against-the-rule (ATR) and oblique (OA).

c) *Sphero-astigmatism*

i) Simple myopic astigmatism (SMA): when one ray comes into focus in front of the retina and one ray comes into focus on the retina. (Plano/ ≥ – 0.25 D).

ii) Compound myopic astigmatism (CMA): occurs when both point of light come into focus in front of the retina. (≥ – 0.5 D / ≥ – 0.25 D).

iii) Hyperopic astigmatism (HPA): occurs when one ray comes into focus in front of the cornea and the other ray comes into focus behind the retina. (+ 0.5 D / ≥ – 0.25 D).

**Emmetropia**: < ±0.50 spherical and < –0.75 cylinder


### Data analysis

Data were analyzed using the Statistical Package for Social Sciences (SPSS) version 21 (SPSS for Windows Inc., Chicago, IL, USA). Descriptive statistics are presented with means and standard deviation while distribution of variables is presented using tables and histograms. Percentages and corresponding 95% confidence intervals are presented as an estimate of all the frequency values. The Kolmogorov-Smirnov (K-S) was used to test for normality of data and data were further analyzed using both normal and non-normal distribution, [both parametric (t-test) and non-parametric Mann-Whitney test respectively] were used to compare means and medians of outcome measures. Fischer’s exact test for small cells less than five samples/data, bivariate logistic regression with Pearson Chi-Squared test was used to analyze percentage differences in URE in relation to demographics between groups. The Student *t-test* was used to analyze differences in means between the headache and control groups respectively. In all analyses, a *p* value of ≤ 0.05 was considered statistically significant.

## Results

### Demographics

The sample demographics are shown in Table 2. Similarities in the sample sizes for both groups were intended to ease statistical analysis as data collection was discontinued when a specific number is reached. Age did not differ significantly between both groups (*p* = 0.45) (Table 2). However, patients aged 19–29 years were most frequent for the HG and 30–40 years for NHG. Females were greater in number than males (*p* = 0.01) (Table 2). The mean age for participants in HG was 25.17 ± 8.66 and 25.32 ± 8.97 for the NHG.

**Table 2:** Sample demographics.


VARIABLES	GROUPS		TOTAL	χ*^2^ (P-VALUE)*

	HEADACHE N = 1062	NO HEADACHE N = 1095		

	Frequency (%)	Frequency (%)		

**Age Group**				1.59 (0.452)

10–18	291 (27.38)	320 (29.22)	611 (27.70)	

19–29	381 (36.73)	380 (34.70)	770 (35.47)	

30–40	381 (35.88)	395 (36.07)	776 (36.83)	

Total	1062	1095	2157	

Mean (SD)	25.17 ± 8.66	25.32 ± 8.97		0.39 (0.700)^µ^

**Gender**				100.99 (**0.001**)*

Male	221 (20.81)	447 (40.82)	668 (30.97)	

Female	841 (79.19)	648 (59.18)	1489 (69.03)	

Total	1062	1095	2157	


Variables with significant associations are asterisked *χ^2^ = Chi-Square; µ = Student t-test; α = Fishers.

In general, the mean and median refractive errors are relatively low in both groups (Table 3).

**Table 3:** Descriptive statistics for refractive variables in headache and non-headache groups.


VARIABLES	HEADACHE	NO HEADACHE		HEADACHE		NO HEADACHE		
		
	MEAN ± SD	MEAN ± SD	S- T-TEST (*P*)	MEDIAN (IQR)	SKEWNESS (KURTOSIS)	MEDIAN (IQR)	SKEWNESS (KURTOSIS)	MANN-WHITN TEST (P)

Sphere	–0.12 ± 1.11	–0.44 ± 2.13	4.31 (0.001)*	0.25 (–0.13–.25)	–4.23 (29.30)	0.0 0(–0.38–0.25)	–6.44 (74.49)	34.14 (0.001*

Cylinder	–0.46 ± 0.52	–0.52 ± 0.69	2.29 (0.021)*	–0.50 (–0.5–0.13)	–2.84 (13.72)	–0.50 (–0.38–0.13)	–3.58 (33.28)	0.19 (0.662)

Myopia	–1.58 ± 1.65	–2.27 ± 3.31	2.88 (0.004)*	–1.0(–1.88–0.50)	–2.84 (12.44)	–1.25 (–2.5–0.50)	–4.75 (33.28)	5.10 (0.024)*

Hyperopia	0.27 ± 0.31	0.25 ± 0.64	1.0 (0.321)	0.35 (0.00–0.63)	3.06 (27.07)	0.25 (0.00–0.63)	9.19 (114.64)	23.89 (0.001

Emmetropia	0.27 ± 0.18	0.23 ± 0.19	4.11 (0.001)*	0.25 (0.25–0.50)	–0.12 (–1.06)	0.25 (0.0–0.50)	0.14 (–1.34)	16.77 (0.001)*

Anisometropia	–0.25 ± 0.89	–0.10 ± 3.10	1.58 (0.133)	–0.25 (–0.37–0.0)	–1.36 (19.31)	–0.25 (–0.37–0.0)	7.80 (208.35)	0.60 (0.487)


### Frequencies of headache types based on location

**Figure 1:**
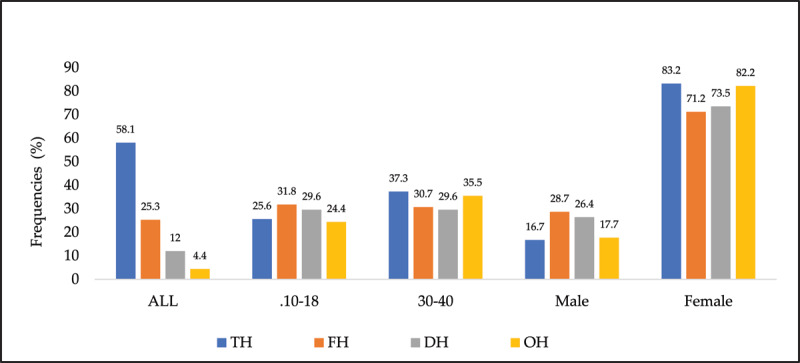
Frequencies of type of headaches and demographics.

**Figure 2:**
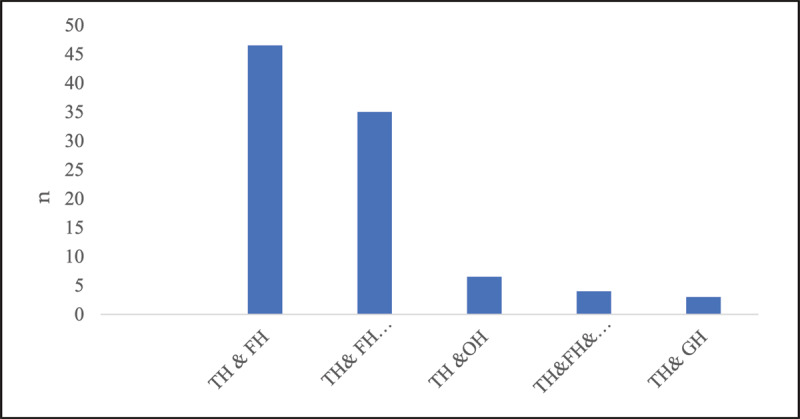
Frequencies of type of headaches.

Temporal and temporo-frontal headaches were significantly the most frequent across all age groups, the OH was the least frequent, and headache types were significantly more frequent in females (Figures 1 and 2).

### Correlations between headache and refractive errors

Hyperopia was the most frequent RE in the HG while anisometropia was most frequent in the NHG (*p* = 0.001) (Table 4).

**Table 4:** Comparing the frequencies of refractive errors and its categories in headache and non-headache groups.


VARIABLE	HEADACHE N = 1058	NO HEADACHE N = 1083	χ^2^ (P-VALUE)	OR (95% CI)	P-VALUE
	
	FREQUENCY (%)	FREQUENCY (%)			

Myopia (All)	226 (21.36)	296 (27.33)	10.03 (0.002)*	0.72 (0.59–0.88)	0.001*

Mild –0.5 to –3.0 DS)	198 (87.61)	241 (81.42)	8.01 (0.018)*	3.77 (1.41–10.12)	0.008*
	
Moderate (–3.25 to –6.0 DS)	23 (10.18)	32 (10.81)		3.31 (1.09–9.99)	0.034*

High (> –6.25 DS)	5 (2.21)	23 (82.14)			

Total	n = 226	n = 296			

Hyperopia (All)	260 (24.57)	211 (19.48)	7.79 (0.005)*	1.35 (1.09–1.65)	0.005*

Mild (0.50–2.0 DS)	258 (99.23)	203 (96.21)	6.74 (0.034)*	–	–
	
Moderate (2.25–4.0 DS)	2 (0.77)	3 (1.42)		NA	NA

High (≥4.25 DS)	0 (0.0)	5 (2.37)			

Total	n = 260	n = 211			

Anisometropia (total)	169 (14.93)	335 (29.18)	36.89(0.001)*	1.88 (1.05–1.33)	0.001*

Astigmatism (All)	204 (19.41)	254 (23.81)	5.88 (0.016)*	0.77 (0.63–0.95)	0.014*

*Amount-astigmatism*					

Mild astigmatism (0.25–0.50 DC)	547 (72.84)	465 (64.67)	11.49 (0.003)*	–	–

Moderate astigmatism (0.75–2DC)	184 (24.50)	227 (31.57)		1.59 (0.88–2.87)	0.125

High astigmatism (>2.0 DC)	20 (2.66)	27 (3.76)		1.09 (0.59–2.01)	0.772

Total	n = 751	n = 719			

*Axis Astigmatism*					

With-The-Rule	302 (40.05)	305 (42.84)	1.06 (0.304)	0.89 (0.72–1.09)	0.315

Against–The-Rule	224 (29.71)	179 (25.14)	3.83 (0.035)*	1.26 (1.03–1.58)	0.050*

Oblique	228 (30.24)	228 (32.02)	0.46 (0.496)	0.92 (0.74–1.15)	

**Total**	n = 754	n = 712			

*Sphero-astigmatism*					

Simple myopic	117 (15.35)	142 (19.32)	4.11 (0.043)*	0.76 (0.58–0.99)	0.043*

Compound myopic	151 (19.82)	198 (26.94)	10.22 (0.001)*	0.67(0.53–0.85)	0.001*

Hyperopic astigmatism	155 (20.34)	113 (15.37)	6.28 (0.012)*	1.41(1.08–1.84)	0.012*

**Total**	n = 423	n = 563			


*Statistically significant (p < 0.05); χ^2^ = Chi-Square; R = Reference; OR = Odds Ratio; NA = ORs could not be generated due to small numbers.

Mild myopia, hyperopia and mild hyperopia, low amount astigmatism (*p* = 0.003), ATR (*p* = 0.003), and hyperopic astigmatism were significantly more frequent than other refractive errors (*p* = 0.012) (Table 4).

### Correlations between headache types and refractive errors

For temporary headaches, total refractive error is significantly higher than emmetropia and TH was significantly the most frequent in anisometropia, hyperopia, mild (low) categories of myopia and astigmatism (Table 5).

**Table 5:** Frequency of headache types in refractive errors and subcategories.


	HEADACHE TYPES							

VARIABLE	TH	χ^2^(P-VALUE)	FH	χ^2^(P-VALUE)	DH	χ^2^(P-VALUE)	OH	χ^2^(P-VALUE)

**Refractive errors**								

Myopia	120 (20.62)	7.46 (0.005)*	46 (18.18)	5.07 (0.16)	35 (29.17)	5.23 (0.156)	11 (24.44)	1.97(0.578)
			
Hyperopia	60 (23.72)		60 (23.72)		26 (21.67)		12 (26.67)	
			
Astigmatism	117 (20.10)		43 (17.0)		20(21.67)		8 (18.18)	
		
Anisometropia (all)	97 (61.0)		34 (21.38)		9 (14.06)		2 (3.13)	

Total Refractive error	489 (84.02)	11.7 (0.008)*	186 (73.52)	0.00 (0.96)	105 (88.24)	2.65 (0.103)	37 (84.09)	0.29 (0.584)

Emmetropia	404 (69.42)		189 (74.70)		76 (63.33)		31 (68.89)	

Severity/amount of myopia& hyperopia								

*Myopia*								

Mild (–0.5 to -3.0DS)	109 (90.83)	0.001*α	40 (86.96)	0.001*α	29 (82.86)	10.58 (0.001)*	187 (88.21)	0.001*α
			
Moderate (–3.25 to –6.0DS)	9 (7.50)		5 (10.87)		6 (17.14)		21 (9.91)	
			
High (> –6.25 DS)	2 (1.67)		1 (2.17)		0 (0.0)		4 (1.89)	

*Hyperopia*								

Mild(0.50–2.0 DS)	148 (99.33)	0.001*α	60 (100.0)	-	26 (100.0)		246 (99.60)	
			
Moderate (2.25–4.0 DS)	1 (0.67)		0 (0.0)		0 (0.0)		1 (0.40)	
			
High (≥ 4.25DS)								

	–		–		–	*–*	–	0.001*α

*Astigmatism*								

Severity/amount								

Low astigmatism (0.25–0.50 DC)	310 (72.60)		125 (74.40)		56 (73.68)	0.001	518 (73.37)	

Moderate astigmatism (0.75–2 DC)	107 (25.06)	241.6 (0.001)	36 (21.43)	95.7 (0.001)	19 (25.0)		169 (23.94)	404.5 (0.001)

High astigmatism (>2 DC)	10 (2.34)		7 (4.17)		1 (1.32)		19 (2.69)	

*Axis astigmatism*								

With-the-rule	165 (38.46)	3.74 (0.154)	67 (39.64)	2.33 (0.31)	35 (46.05)	4.82 (0.090)	20 (57.14)	6.28 (0.043)

Against-the-rule	132 (30.77)		49 (28.99)		17 (22.37)		8 (22.86)	

Oblique	132 (30.77)		53 (31.36)		24 (31.8)		7 (20.0)	

*Sphero-astigmatism*								

Simple myopic	64 (29.78)	3.59 (0.166)	32 (32.65)	0.19 (0.90)	6 (16.67)	3.74 (0.155)	5 (23.81)	0.67 (0.716)
			
Compound myopia	86 (35.98)		31 (31.63)		14 (38.89)		8 (38.10)	

Hyperopic	89 (37.24)		35 (35.71)		16 (44.44)		8 (38.10)	


Frontal headaches, DH and OH were most frequent in mild -myopia and -astigmatism while OH was significantly most frequent in WTR astigmatism (Table 5). Most headache types were mostly common in hyperopic astigmatism although some were not significantly so (Table 5).

**Figure 3:**
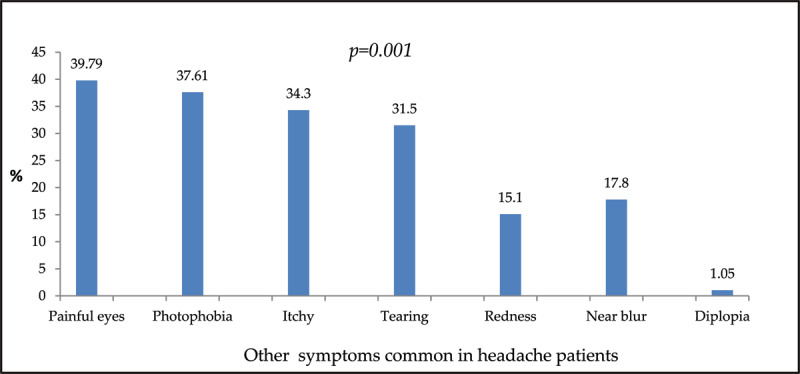
Symptoms associated with headache.

As shown in Figure 3, painful eyes were most frequent among headache patients (Figure 3).

## Discussion

This study reported and investigated the frequencies of, and associations among, refractive errors and headaches in patients aged between 10 and 40 years examined at an independent optometry practice. The major findings include that low-amount myopia, hyperopia, and astigmatism, ATR astigmatism and hyperopic astigmatism were significantly more frequent in the headache group (Table 4). Temporal and temporo-frontal headaches were the most frequent headache locations. On the distribution of headache types in uncorrected refractive errors, TH was significantly the most frequent in anisometropia, mild severities of hyperopia, myopia, and astigmatism. Findings from this study suggest that patients who have low spheres and cylindrical errors as well as ATR astigmatism are more likely to experience headaches than patients who do not have such anomalies. This suggests that these errors may be risk factors or triggers for the headache that patients experience. These findings are clinically relevant given the burden of headaches and its effect in visual efficiency. Findings are discussed in context with previous studies based on the study objective-as frequency and association of refractive errors and headaches, gaps in knowledge in the context of study designs, mechanisms and existing theories with the goal to improve the validity of future studies.

In the present study, the frequencies of uncorrected refractive errors among headache patients were: hyperopia 24.5%, myopia 21.3%, astigmatism 19.4%, and anisometropia 14.9% (Table 4). These frequencies fall within the frequency ranges reported in previous studies, which included myopia 12–35.7%, hyperopia 12–31% while astigmatism was 33%–43.9% (Appendix A). Furthermore, mild hyperopia, low amount astigmatism, ATR, and hyperopic astigmatism were significantly more frequent in the headache group. Clinical experience, findings from present study and narrative reports (Bellow 1968; Grosvenor 2007; Gordon 1966) indicate that low spherical and cylindrical errors tend to be more associated with asthenopia than do high-amount errors. However, empirical studies to support the hypotheses are few (Thorud et al. 2021; Marasini et al. 2012).

On the distribution of headache types in refractive errors, all types were frequent in low spherical and cylindrical errors, as well as anisometropia (Table 5). Singh, Mishal & Sunarait (2021) found significant association between ‘location of headache and ocular causes of headache’ although they did not give much details. Overall, findings differed markedly across studies due to variations in study designs (Appendix A).

Hyperopia is the most frequent refractive error among headache patients in the present study and agrees with other studies (Akinci et al. 2008; Das & Gupta 2017; Lajmi et al 2021; Gil-Gouveia & Martins 2002; Rydberg 2005; Wajuihian 2015; Vilela et al. 2015; Thorud, et al. 2021; Siqueira et al. 2021). Hyperopia has consistently been associated with eyestrain/headache (Grosvenor 2007; Solan 1990; Rydberg 2005; Hendricks et al. 2007). Hyperopia is also associated with reduced reading ability, and poor school performance (Solan 1990). Specifically, low-amount hyperopia may cause intermittent blur, fatigue, loss of concentration, and inattention in some children (Grosvenor 2007; Solan 1990).These symptoms may be mistaken for a short attention span (Grosvenor 2007; Solan 1990). Symptoms in uncompensated hyperopia results from excessive accommodative demand in hyperopia.

The significant association between astigmatism and headaches found in the present study agrees with several studies (Lajmi et al. 2021; Das & Gupta 2017; Hendricks et al. 2007; Singh, Mishal & Sunarait 2021; Sardar, Chaudhary, & Dubey 2020; Prabhu & Raju 2016; Sharma, Sharma & Kai 2021; Padha & Qayum 2019). In astigmatism, the axis, amounts and spherical components influence asthenopia as visual acuity and visual perception (Grosvenor 2007; O’Leary & Evans 2003) are important for visual efficiency (Wajuihian 2015; O’Leary & Evans 2003). In hyperopic astigmatism, accommodation may improve distance and near vision at the expense of feeling of eyestrain. However, in simple or compound myopic astigmatism, there could be blurred vision at distance, although at near viewing, accommodation may place the circle of least confusion closer to-or on the retina, depending on the amount of astigmatism and thus induce headaches (Grosvenor 2007; Wajuihian 2015; O’Leary & Evans 2003).

Regarding amount-astigmatism, with high astigmatism, headaches may be minimal because the ciliary muscles makes minimal efforts to correct such astigmatic error. (Grosvenor 2007; Wajuihian 2015; Cameron 1976). However, with low astigmatic error, the patient makes unconscious efforts to compensate for the defect. Although visual acuity could be normal for small astigmatic errors; if either focal line temporarily focuses onto the retina, accommodation becomes unstable, and results in asthenopia (O’Leary & Evans 2003; Cameron 1976). Sometimes the efforts are excessive and cause the ciliary muscle to contract irregularly, altering the lenticular and corneal astigmatism dynamics such as changing WTR to ATR astigmatism. This process results in more headaches (O’Leary & Evans 2003; Cameron 1976). Therefore, low-amount astigmatism especially with ATR may cause more severe headaches than does a high amount (O’Leary & Evans 2003; Cameron 1976).

Anisometropia was significantly associated with temporal headaches in the present study (Table 5). Padha & Qayum 2019, Akinci et al 2008, & Siqueira et al, 2021 found significant association between headaches and anisometropia although they did not specify the headaches anatomic location. Anisometropia has been reported to cause more headaches because of the difference in error between both eyes and due to the extra effort to accommodate to a different extent between both eyes (Grosvenor 2007). Myopia was significantly associated with headache in some studies (Singh, Mishal & Sunarait 2021; Rao, Ponnada & Thilagavathi 2015). Headaches in myopia results from excessive squinting of the forehead and eyelids in an effort to narrow the palpebral fissure and using the pinhole effect to see better (Grosvenor 2007; Dotan, Stolovitch & Moisseiev 2014). A few studies (Roth et al. 2014; Cameron 1976; Al-Rasheed 2020; Abolbashariet al. 2014) found no significant association between refractive errors and headaches. Another theory on the source of headache is that postural adaptations while sitting may strain the neck muscles, which may cause headache as a form of myofascial referred pain, or tension-type headache (Melis 2003).

A notable finding in the present study is that the high frequencies of headache were accompanied by relatively high frequencies of other symptoms including photophobia, itchiness, tearing, and tired and painful eyes (Figure 4). This finding was also reported in some studies (Wajuihian 2015; Mvitu & Kaimbo 2003; Roth et al. 2014). The mechanism of such coexistence of headaches and associated symptoms was explained by Grosvenor (2007). Grosvenor (2007) proposed that through an extensive neural mechanism, the triad of pain, photophobia, and tearing respond to the stimulation of the ophthalmic division of the fifth cranial nerve at multiple anatomical levels from the eye to the cortex.

Consequently, the local axon reflex is activated in an attempt to compensate for an anomaly, which in turn stimulates the sensory nerve endings. This process results in a local increase in blood supply, which may manifest as hyperemia of the conjunctiva or lid (Grosvenor 2007). The resulting hyperemia creates the sensations of tearing, itching and pain, and causes the individual to rub the eyes (Grosvenor 2007).

Temporal and temporo-frontal headaches being the most frequent headache types agrees with some studies (Thorud et al. 2021; Roth et al. 2014) and contrast with other studies which found frontal headaches to be more frequent (Marasini et al. 2012; Singh, Mishal & Sunarait 2021)., In general, refractive errors may produce headaches and pains in the frontal, bi-temporal, and occipital regions and at the back of the neck (ICHD 2013; Cameron 1976).

Ocular headache of functional cause is an acute or chronic discomfort that results from prolonged performance of near task. Ocular headache is a reflex (referred) pain, which results from stimulation of the endings of the nasal branch of the ophthalmic division of the fifth cranial nerve (Cameron 1976; Wajuihian 2015).

Functional ocular headache (eyestrains) may be acute or chronic and results from the prolonged performance of near task. The eyestrain is caused by the sustained contraction of the ciliary muscles with vascular engorgements (Grosvenor 2007; Cameron 1976). The extrinsic eye muscles or the occipito-frontalis may also be involved in the mechanism of various types of headache (Grosvenor 2007; Cameron 1976). With the performance of pronged near task, ocular headaches result from the stimulation of the endings of the nasal branch of the ophthalmic division of the fifth cranial nerve. Being a reflex (referred) pain, ocular headache radiates to any part of the area of distribution of the first division of the fifth cranial nerves (Grosvenor 2007; Cameron 1976; Wajuihian 2015). The pain may manifest at various times of the day but mostly in the late afternoons or early evenings-at the end of a day’s work (Grosvenor 2007; Cameron 1976).

On demographics and headaches, females had higher frequency of headaches. In adolescents-females especially, headaches seem to increase with the transition to adolescence and in relation to psychosocial stress and obesity (Thorud et al. 2021). Females’ preponderance of headaches agrees with other studies (Lajmi et al. 2021; Marasini et al. 2012; Hendricks et al. 2007; Gordon 1966).

The highest frequency of headache was found in age group 19-29 years, which agrees with other studies (Jain et al. 2015; Singh, Mishal & Sunarait 2021; Supriya & Nakkella 2021), may be related to increased near-task demand with an increase in academic or near task responsibilities among patients of that age group.

### Implications, limitations and strengths

The findings of this study are relevant given the roles that the refractive mechanism play in vision efficiency during near tasks, given that these low errors are sometimes overlooked by clinicians or outright denied (Roth et al. 2014; Gil-Gouveia & Martins 2002).

A possible limitation of the study is that the relation of headaches and diet, sleep pattern, medication, other health conditions, which include smoking, alcohol consumption, was not reported although they are not part of this study.

The strengths of this study include its relatively firm prospective design, novelty and the heterogeneous nature of the patients-base. Furthermore, the findings have implications and can be applied in clinical practice and research in vision care as well as will guide future studies.

## Recommendations

These recommendations outlined below will improve validity of future studies

- Cycloplegia reveals the full extent of refractive errors and should be applied especially in studies, which enrol children.
- The use of spherical equivalent as was the case in some previous studies (Appendix A) could influence the study outcomes when individual refractive error is related to symptoms thus the use of absolute values should be used instead of SE.
- Further studies with larger sample size, prospective design with random sampling is recommended. Adopting prospective design will minimise the limitations associated with retrospective study design.
- Adequate scientific rigours should be applied to the conduct of future studies. In addition, for consistent comparison of findings, participants of a specific age range should be studied. In that case, studies on school-aged children should be specific to that age range of approximately six to 18 years.
- Overall, using a standard study protocol across studies on headache and refractive errors is more likely to yield more valid and useable outcomes for policy administration, research and clinical practice. Such standardised protocol is not available.
- Future study, which will include occupation of sample groups to assess any relation with headache type and activity, will be more useful.

## Conclusion

The present study provides new data, which were not previously available, and advances knowledge on headaches and refractive errors.

Temporal and temporo-frontal headaches were the most frequent types. Patients with mild types of hyperopia and astigmatism (especially against the rule astigmatism) are significantly more likely to present with headaches than those without such refractive errors and vice versa. A major motivating factor for patients to consult the optometrist is the headache they experience. Although headache may be a symptom of low uncorrected hyperopia or astigmatism, it has not been proven to be caused by refractive error. However low refractive error need be corrected when symptomatic.

## Additional File

The additional file for this article can be found as follows:

10.22599/bioj.313.s1 Appendices. Appendix A to B.

## References

[1] Abolbashari, F, Hosseini, SMA, AliYekta, A and Khabazkhoob, MN. 2014. The correlation between refractive errors and headache in the young adults. Austin J Clin Ophthalmol, 1(3): 1014.

[2] Al-Rasheed, SH. Clinical characteristics of patients presenting with headache at binocular vision clinic: a hospital based study: 2020. Pak J Ophthalmol, 36(3): 247–252. DOI: 10.36351/pjo.v36i3.1046

[3] Akinci, A, Güven, A, Degerliyurt A, Kibar, E, Mutlu, M and Citirik, M. 2008. The correlation between headache and refractive errors. J AAPOS, 12(3): 290–3. Epub 2008 Mar 10. PMID: 18329921. DOI: 10.1016/j.jaapos.2007.11.018 18329921

[4] Bellows, JG. 1968. Headahes and the eye. Headache, 7(4): 165–170. DOI: 10.1111/j.1526-4610.1968.hed0704165.x 5635165

[5] Bigley, GK. Headache. In: Walker, HK, Hall, WD and Hurst, JW, Clinical Methods: The History, physical, and laboratory examinations. 3rd ed. Boston: Butterworths; 1990. Chapter 54. PMID: 21250218. 21250045

[6] Cameron, ME. 1976. Headaches in relation to the eyes. Med J Aust, 1(10): 292–4. DOI: 10.5694/j.1326-5377.1976.tb115293.x 1272098

[7] Christopher, J, Priya, Y, Bhat, V and Sarma, G. 2022. Characteristics of headache in children presenting to ophthalmology services in a tertiary care center of South India. Cureus, 14(2): 24–9 e21805. PMID: 35251869; PMCID: PMC8890450. DOI: 10.7759/cureus.21805 PMC8890450 35251869

[8] Das, D and Gupta, S. 2017. A study on refractive errors in schoolchildren with complaints of headache in a rural tertiary care hospital. Ind J Clin & Expt Ophth, 3(2): 192.

[9] Donahue, HC. 1958. Some current concepts of headache, especially ocular. AMA Arch Ophthalmol, 59(4): 489–94. DOI: 10.1001/archopht.1958.00940050045003 13519967

[10] Dotan, G, Stolovitch, C and Moisseiev, E. 2014. Uncorrected ametropia among children hospitalized for headache evaluation: a clinical descriptive study. BMC Pediatr, 14: 1–4. DOI: 10.1186/1471-2431-14-241 25266370 PMC4190346

[11] Gil-Gouveia, R and Martins, IP. 2002. Headaches associated with refractive errors: myth or reality? Headache, 42(4): 256–62. PMID: 12010381. DOI: 10.1046/j.1526-4610.2002.02077.x 12010381

[12] Gordon, DM. 1966. Some headaches in an ophthalmologist’s office. Headache, 6(3): 140–45. DOI: 10.1111/j.1526-4610.1966.hed0603141.x 5918291

[13] Grosvenor, T. 2007. Primary Care Optometry. 5th ed. Philadelphia: Butterworth Heinemann Elsevier.

[14] Hendricks, TJ, Brabander, J, van Der Horst, FG, Hendrikse, F and Knottnerus, JA. 2007. Relationship between habitual refractive errors and headache complaints in schoolchildren. Optom Vis Sci, 84(2): 137–43. PMID: 17299344. DOI: 10.1097/OPX.0b013e318031b649 17299344

[15] Hale, N and Paauw, DS. 2014. Diagnosis and treatment of headache in the ambulatory care setting: a review of classic presentations and new considerations in diagnosis and management. Med Clin North Am, 98(3): 505–27. DOI: 10.1016/j.mcna.2014.01.006 24758958

[16] International Classification of Headache disorders (ICHD). The international classification of headache disorders, 3^rd^ edition (beta version). Cephalalgia, 2013; 33: 629–08. DOI: 10.1177/0333102413485658 23771276

[17] Jain, S, Chandravanshi, SL, Dukariya, L, Tirkey, ER and Jain, SC. 2015. Clinical study of headache with special reference to ophthalmic cause. Int J Med Sci Public Health, 4(2): 292–97 DOI: 10.5455/ijmsph.2015.1910201454

[18] Jain, SA, Sutapa, D, Subashini, M and Mahadevan, K. 2018. Determination of the proportion of refractive errors in patients with primary complaint of headache and the significance of refractive error correction in symptoms relief. Ind J Clin & Expt Ophth, 4(2): 258-262 DOI: 10.18231/2395-1451.2018.0057

[19] Khatatbeh, AE, Othman, EF, Alalawneh, AM, Albdour, MQ, Jaradat, TF, Al Hazaimeh, AM, Ahmed, M and Abbas, K. 2021. Ocular and dental causes of headaches among school-age children in Jordan: A retrospective study. Cureus, 13(6): e15623. PMID: 34277240; PMCID: PMC8277091. DOI: 10.7759/cureus.15623 34277240 PMC8277091

[20] Kondam, R and Settypalli, RR. 2017. A study to assess causes of headache in ophthalmic practice in a tertiary care teaching hospital – A hospital based cross-sectional study. MedPulse Intl J of Ophthal, 3(2): 45–7. Available from: https://www.medpulse.in/Ophthlmology/

[21] Lajmi, H, Choura, B, Achour, B, Doukh, Z and Amin, W. 2021. Headache associated with refractive errors: Characteristics and risk factors. Revue neurologique, 188(8): 947–54. DOI: 10.1016/j.neurol.2020.10.008 33483090

[22] Marasini, S, Khadka, J, Sthapit, PR, Sharma, R and Prasad, BJ. 2012. Ocular morbidity on headache ruled out of systemic causes: A prevalence study carried out at a community based hospital in Nepal. J Optom, 5(2): 68–74. DOI: 10.1016/j.optom.2012.02.007

[23] Mehboob, MA, Nisar, H and Khan, M. 2019. Ametropia in children with headache. Pak J Med Sci, 35(3): 701–04. DOI: 10.12669/pjms.35.3.268 31258579 PMC6572967

[24] Melis, M. 2003. Headache associated with refractive errors: overestimated or overlooked? Headache, 43: 297–8. PMID: 12603655. DOI: 10.1046/j.1526-4610.2003.03060.x 12603655

[25] Mvitu, MM and Kaimbo, WK. 2003. Manifestations of asthenopia in black subjects. Bull Soc Belge Ophthal, 289: 45–49. 14619629

[26] O’Leary, C and Evans, BJW. 2003. Criteria for prescribing optometric interventions: Literature review and practitioner survey. Ophthal Physiol Opt, 23(5): 429–39. DOI: 10.1046/j.1475-1313.2003.00137.x 12950889

[27] Parajuli, S, Shrestha, R, Chapagain, S, Shrestha, R, Singh, P and Acharya, S. 2021. Ocular causes of headache in patients presenting to a sub-urban eye hospital. J Ophthalmol Adv Res, 2(1): 1–9. DOI: 10.46889/JOAR.2021.2105

[28] Padha, A and Qayum, S. 2019. The prevalence of refractive errors in patients presenting with headache. Global J Rs anal, 8(6): 1–3e.

[29] Philipp, J, Zeiler, M, Wöber, C, Wagner, G, Karwautz, A and Steiner, TJ. 2019. Prevalence and burden of headache in children and adolescents in Austria – a nationwide study in a representative sample of pupils aged 10–18 years. J headache & pain, 20(1): 1050–101. DOI: 10.1186/s10194-019-1050-8 PMC6836380 31694547

[30] Prabhu, PB and Raju, KV. 2016. Role of refractive errors in inducing asthenopic symptoms among spectacle corrected in ammetropes. BMH Med Jour, 3(2): 32–6. Available at: https://www.babymhospital.org/BMH_MJ/index.php/BMHMJ/article/view/85>.

[31] Rao, CM, Ponnada, S and Thialgavathi, R. 2015. Assessment of opthalmologicsl causes of headaches in a tertiary care center in South India. Int J Sc stud, 2(10): 90–93.

[32] Roth, Z, Pandolfo, KR, Simon, J and Zobal-Ratner, J. 2014. Headache and refractive errors in children. J Pediatr Ophthalmol Strabismus, 51(3): 177–9. PMID: 24804974. DOI: 10.3928/01913913-20140429-02 24804974

[33] Rydberg, A. 2005. Asthenopia in schoolchildren, Orthoptic and ophthalmological findings and treatment. Doc Ophthal, 111(2): 65–72. DOI: 10.1007/s10633-005-4722-4 16514487

[34] Sardar, A, Chaudhary, AK and Dubey, AK. 2020. Correlation between headache and refractory error in school children with complaints of headache in a tertiary health care hospital. IOSR Journal of Dental & Medical Sciences, 19(4): 24–26.

[35] Sharma, P, Sharma, A and Kai, S. 2021. Refractive errors and headache: a clinical study among patients attending ophthalmology OPD in a tertiary care hospital. European Journal of Molecular & Clinical Medicine, 8(4): 2362–66.

[36] Singh, P, Mishal, A and Sunarait, JS. 2021. Ophthalmic causes of headache among patients attending tertiary care center in Kathmandu, Nepal. Nepal Med Coll J, 23(3): 235–40. DOI: 10.46889/JOAR.2021.2105

[37] Siqueira, PT, Valença, LP, Andrade, JR and Valença, MM. 2021. Pediatric patients at a high risk or headache of ocular origin: the HAMS Score (Hyperopia, Astigmatism, Myopia, and Strabismus) Headache Med, 12(2): 134–40. DOI: 10.48208/HeadacheMed.2021.24

[38] Solan, H. 1990. Learning disabilities. In: Rosenbloom, A and Morgan, MW (eds.), Principles and practice of pediatric optometry. New York: JP Lippincott n.p.

[39] Supriya, BN and Nakkella, N. 2021. To Determine The Ophthalmic Causes Of Headache In Patients Attending Tertiary Care Centre Eye OPD At Tumkur. Int J Health & Clin l Research, 4(10): 138–41. Available at: https://ijhcr.com/index.php/ijhcr/article/view/1617.

[40] Thorud, HM, Aurjord, R and Falkenberg, HK. 2021. Headache and musculoskeletal pain in school children are associated with uncorrected vision problems and need for glasses: a case–control study. Sci Rep, 11(1): 2093–011. DOI: 10.1038/s41598-021-81497-w 33483534 PMC7822909

[41] Vilela, MA, Castagno, VD, Meucci, RD and Fassa, AG. 2015. Asthenopia in schoolchildren. Clin Ophthalmol, 28(9): 1595–603. PMID: 26357460; PMCID: PMC4559242. DOI: 10.2147/OPTH.S84976. PMC4559242 26357460

[42] Wajuihian, SO. 2015. Frequency of asthenopia and its association with refractive errors. African Vision and Eye Health, 74(1): 2–11. DOI: 10.4102/aveh.v74i1.293

[43] Wajuihian, SO. 2022. Characterising accommodative and vergence anomalies and symptoms in an optometry clinic population. Br Ir orthop J, 18(1): 76–92. DOI: 10.22599/bioj.267 PMC9284986 35903147

[44] Wajuihian, SO and Mashige, KP. 2021. Gender and age distribution of refractive errors in an optometric clinical population. J Optom, 14(4): 315–27. Epub 2021 PMID: 33487574. DOI: 10.1016/j.optom.2020.09.002 33487574 PMC8569398

