# Supplementary material for: Exploring Correlations between Headaches and Refractive Errors in an Optometry Clinic Sample
Source: Br Ir Orthopt J. 2024 Jan 3;20(1):1–15. doi: 10.22599/bioj.313 (PMC10768566; doi:10.22599/bioj.313)
Supplement: Appendices. — Appendix A to B. [file bioj-20-1-313-s1.pdf]

LIST OF APPENDICES

Appendix A: Summary of previous studies on frequency

	**Age range studied**	**Sample size**	**Cycloplegia Yes/no**	**Country of study**	**Study design Retro/prospective**	**Myopia Cut off /%**		**Hyperopia Cut off /%**		**Astigmatism Cut off /%**		**Anisometropia Cut off /%**		**LM**	**MM**	**HM**	**LH**	**MH**	**HH**	**LA**	**MA**	**HA**
**Present study**	10-40	1065	yes	South Africa		≤0.50	1.3	≥0.50	24.5	≤0.75	19.4	± 0.75	14.9	87.6	10.1	2.21	99.2	0.7	0	72.8	24.5	2.6
Thoroud et al. (2021)	10-15	20	yes		Non-clinic –based. Prospective cross-sectional	≤ 0.50	5	0.50-2.0	65	≤0.75	10	≥1.00	0									
Dotan et al. (2014)	8-18	917	yes		Clinic/retrospective	≤ 0.50 SE	63	2	38	≤1.00	19	≥1.00										
Siqueira, Valença, Andrade, (2021)	0-19	726	no	Brazil	Clinic/retrospective																	
Shazia & Qayum	8 -18 years	155	yes	India	Clinic/prospective																	
Sardar, Chaudhary, Dubey (2020)	8-17	402	no	India	School/prospective	Not reported	13.7		26.6		59.6											
Abolbashari et al (2014)	21.02 ± 1	74	no		Non/clinic-Based prospective	≤ 0.50	27.0	≥ 0.50	45	≤0.50	40	NR	NR	20.8	62.5	0	45.4	0	0			
Das & Gupta (2017)	10-18	238	no		Prospective/clinical	SE ≤ 0.50	12.3	≥ 0.50	26.8	≤0.50	60.6	NR	NR	NR	NR	NR	NR	NR	NR	NR	NR	
Singh et al (2021)	18-61	132	no		Clinic/prospective	NR	33.4	NR	NR		36.5	NR	NR	NR	NR	NR	NR	NR	NR	NR	NR	
Supriya &Nakkella (2021)	16-45	180	yes		Clinic/prospective	NR	NR	NR	28.8	NR	35.8	NR	NR	NR	NR	NR	NR	NR	NR	NR	NR	
Qayum& Padha (2019)	8 -18	155	yes		Clinic/prospective	≤0 .50	6.6	≥ 2.00	7.6	≤1.00	24.7		23.8									
Christopher et al (2021)	10-18	311	Not reported		Clinic/prospective	NR	75.3	NR	14.6	NR	10.1											
Kondom et al (2017)	5-52	315	no		Clinic/prospective	NR	23	≥1	14	N/R												
Parajuli et al (2021)	37.1	200	yes		Clinic/prospective	≤ 0.50	17.3	≥ 0.50	13.0.	≥0.50	69.5											
Hendricks et al (2007)	11-13	487	no		School/propsective	SE ≤ 0.50	15	≥ 0.50	12	≤0.25	33											
Lajmi et al (2021)	24.8 ± 11.2	50	no		Clinic/retrospective	SE ≤ 0.50	12	≥ 0.50	22	≤0.50	41											
Mahboob et al (2019)	5-16	262	yes		Clinc /prospective	NR	35.7	NR	21.50	NR	42											
Akinci et al (2008)	8-18	310	yes		Clinic/retrospective	SE ≤0.50	6.5	≥ 2.00	8%	≤1.00	19.6											
Khatabeh et al (2021)	5-13	712	yes		Clinic/retrospective	SE ≤ 0.50	34.2	≥ 0.50	21.9	≤0.75	43.9											
Jain et al (2015)	16-30	1520	yes		Clinc /prospective	NR	12.4	NR	21.5	NR	42	25										
Jain et al (2018)	6-36	103	yes		Clinc /prospective	≤ 0.50	20.6	≥ 0.50	31.03	≥0.50	**NR**											
						**Range 12-35.7%**			**12-31.0**	**33-43.9**												

Appendix B: Summary of previous studies on frequency

Authors	Age range (years)	Sample size	WTR	ATR	Oblique	Simple	Compound	Hyperopic	Mixed
**Present study**	1065	1065							
Thoroud et al, (2021)	20	20							
Dotan et al, (2014)	917	917							
Gil Gouveian et al (2002)	Mean age 37.6	105 main							
Abolbashari et al (2014)	Mean age 21.02 ± 1	74	40	33	62				
Das & Gupta (2017)	10-18	238	31	82	26 (Count)				
Singh et al (2021)	18-61	132				22.8	0.8		
Supriya &Nakkella (2021	16-45	180	40.2	9.8	4.9	42	9.8	4.9	45.1
Akinci et al (2008)	8-18	6-36				1.6	7.7		10
Jain et al (2015)	6-36	1520				6.90	13.7	10.3	17.2,
Jain et al (2018)	16-30	103				14.5	6.5	16	1
Christopher et al (2021)	10-18	311							
Parajuli et al (2021)	37.1	200							
Hendricks et al (2007)	11-13	487							
Lajmi et al (2021)	712	50							
Mahboob et al (2019)	5-16	16-30							
Khatabeh et al (2021)	5-13	712							

Appendix C: Frequency of headaches types and subtypes

Variable	All n % [95%CI]	Age Groups			χ^2*(p-value)*	Sex		χ^2 *(p-value)*
		**10-18**	**19-29**	**30-40**		**Male**	**Female**	
TH	584 (58.17)[55.04-61.23]	150 (25.68)	216 (36.99)	218 (37.33)		98 (16.78)	486 (83.22)	
FH	254 (25.30)[22.66-28.13]	81 (31.89)	95 (37.40)	78 (30.71)	6.09 (0.41)	73 (28.74)	181 (71.26)	17.87**(0.005)***
DH	121 (12.05)[10.14-14.26]	35 (29.66)	38 (32.20)	45 (38.14)		32 (26.45)	89 (73.55)	
OH	45 (4.48)[3.32-6.0)]	11 (24.44)	18 (40.0)	16 (35.56)		8 (17.78)	37 (82.22)	
**Combined headache types**								
TH+FH	93 (46.5)[39.44-53.67]	23 (24.73)	29 (31.18)	41 (44.09)		15 (16.13)	78 (83.87)	
TH+GH	6 (3.0)[1.11-6.42]	1 (16.67)	1 (16.67)	4 (66.67)		2 (33.33)	4(66.67)	
TH+OH	13 (6.5)[3.51-10.86]	1 (7.69)	4 (30.77)	8 (61.54)	0.229$^{\alpha}$	1 (7.69)	12 (92.31)	0.115$^{\alpha}$
TH+FH+GH	2 (1.0)[0.12-3.57]	1 (50.0)	1 (50.0)	0 (0.0)		0 (0.0)	2 (100.0)	
TH+FH+OH	70 (35.0)[28.41-42.05]	21 (30.0)	30 (42.86)	19 (27.14)		12 (17.14)	58 (82.86)	
TH+FH+OH+GH	8 (4.0)[1.74-7.73]	2 (25.0)	4 (50.0)	2 (25.0)		4 (50.0)	4(50.0)	
FH+OH	6 (3.0)[1.11-6.42]	2 (33.33)	3 (50.0)	1 (16.67)		1 (16.67)	5(83.33)	
